# Molecular Characterization and Screening for Sheath Blight Resistance Using Malaysian Isolates of *Rhizoctonia solani*

**DOI:** 10.1155/2014/434257

**Published:** 2014-08-28

**Authors:** Kalaivani Nadarajah, Nurfarahana Syuhada Omar, Marhamah Md. Rosli, Ong Shin Tze

**Affiliations:** ^1^School of Environmental and Natural Resources Sciences, Faculty of Science and Technology, Universiti Kebangsaan Malaysia, 43600 Bangi Selangor, Malaysia; ^2^School of Bioscience and Biotechnology, Faculty of Science and Technology, Universiti Kebangsaan Malaysia, 43600 Bangi Selangor, Malaysia

## Abstract

Two field isolates of *Rhizoctonia solani* were isolated from infected paddy plants in Malaysia. These isolates were verified via ITS-rDNA analysis that yielded ~720 bp products of the ITS1-5.8S-ITS4 region, respectively. The sequenced products showed insertion and substitution incidences which may result in strain diversity and possible variation in disease severity. These strains showed some regional and host-specific relatedness via Maximum Likelihood and further phylogenetic analysis via Maximum Parsimony showed that these strains were closely related to *R. solani* AG1-1A (with 99-100% identity). Subsequent to strain verification and analysis, these isolates were used in the screening of twenty rice varieties for tolerance or resistance to sheath blight via mycelial plug method where both isolates (1801 and 1802) showed resistance or moderate resistance to Teqing, TETEP, and Jasmine 85. Isolate 1802 was more virulent based on the disease severity index values. This study also showed that the mycelial plug techniques were efficient in providing uniform inoculum and humidity for screening. In addition this study shows that the disease severity index is a better mode of scoring for resistance compared to lesion length. These findings will provide a solid basis for our future breeding and screening activities at the institution.

## 1. Introduction

*Rhizoctonia solani* Kühn (teleomorph:* Thanatephorus cucumeris* (Frank) Donk) is a soil-borne pathogen that causes a wide variety of diseases in economically important crop species. The genus* Rhizoctonia* in the anamorphic classification is divided into 3 main group forms: multinucleate* Rhizoctonia* (teleomorphs:* Thanatephorus* and* Waitea*), binucleate* Rhizoctonia* (teleomorphs:* Ceratobasidium* and* Tulasnella*), and uninucleate* Rhizoctonia* (teleomorph:* Ceratobasidium)* [1, 2].* Rhizoctonia solani,* a member of the multinucleate* Rhizoctonia* group [3], is a genetically diverse causal agent of rice sheath blight in many developing countries. This organism has resulted in major constraint of rice production over the past two decades [4, 5]. The anastomosis group AG 1 and the subgroup IA have been implicated in causing infections in rice [6–8].

*R. solani* can be divided into 13 anastomosis groups (AGs) based on hyphal anastomosis reaction [9]. Some AGs of* R. solani* have been further divided into subgroups based on cultural morphology, host range, virulence, and molecular techniques [10]. Isolates within the same AG, or within the same subgroup, may have similar characteristics, such as host preference and disease symptoms. Currently the AG groups of* Rhizoctonia solani* have been further divided into subgroups AG 1, AG 2, AG 3, AG 4, AG 5, AG 6, AG 7, AG 8, AG 9, AG 10, AG 11, AG 12, and AG 13 based on various features [11, 12]. As of recent, it has become increasingly common to designate anastomosis groups through molecular approach.

Most available molecular techniques are based on the detection and typing of genomic polymorphisms at several levels. Sequencing of ribosomal DNA (rDNA) has been widely employed in recent years to reconstruct phylogenetic relationships between different organisms at the genus level [13, 14]. Techniques in molecular biology have contributed to determining genetic diversity and taxonomic classification within fungal species [15, 16]. Currently, rDNA internal transcribed spacer region ((ITS) composed of ITS1, 5.8S, and ITS2 regions) sequence information offers the most accurate method of establishing the taxonomic and phylogenetic relationships for* Rhizoctonia* spp. [2, 8]. Sequence analysis of the genomic regions encoding the ITS-rDNA is convenient for AG determination and has become increasingly common with the accumulation of sequences from different isolates in databases [17, 18]. Both within and between the various AGs, ITS-rDNA sequencing has been used to analyze the genetic diversity of* R. solani*. The phylogenetic relationships of a number of fungi, including phytopathogenic fungi, have been studied through the rDNA sequences. In a previous study of* R. solani,* ITS-rDNA regions and rRNA genes were analyzed by a combination of PCR-amplification and RFLP. This study revealed a genetic divergence within AG-1 or AG-2 [19, 20] based on patterns of ITS-rDNA variation and characterized the evolutionary relationships among AGs based on DNA polymorphism in the 18s and 28s rRNA gene regions [8, 21].

Modulating control of diseases caused by this phytopathogen requires a better understanding of the organism. Blight causes up to a 50% decrease in the rice yield every year under favourable conditions worldwide [22–24]. In Eastern Asia, it affects approximately 15 to 20 million ha of irrigated rice and causes yield losses of up to 6 million tons of rice per year [23]. The initial symptoms of this disease include lesions on the sheaths of lower leaves at late tillering or early internode elongation growth stages and may coalesce to encompass the entire leaf sheath and stem [6, 25, 26]. Thus far, only partial resistance to rice sheath blight has been identified, as seen with the screening of thousands of rice cultivars from various rice growing regions that yielded no cultivar exhibiting resistance [4, 12].

According to Eizenga et al. [27], the wild* Oryza* species which are closely related to cultivated rice are a potential source of important traits including new resistance genes to fight pests like sheath blight and blast diseases. Some examples of wild varieties that have been used in transferring resistance to cultivated rice through backcrossing are* O. minuta* J.S. Presl. ex C.B. Presl. and* O. officinalis* Wall ex Watt [28]. Other researchers have shown that* Oryza spp*. P346356,* O. nivara* P560409,* O. rufipogon* P590420, and* O. barthi* 1237987 were highly susceptible to* R. solani* infections while Jasmine 85 P1595927,* O. officinalis* IRGC105979, and* O. meridionalis* IRGC105306 were reported to be moderately resistant [29]. In this research, we included two of our own ABQTL lines (UKMRC2 and UKMRC9) and MR219 (a local cultivated rice variety) in our screening studies to identify potential donor lines. UKMRC2 and UKMRC9 were generated from a backcross between* Oryza rufipogon*, a wild rice and MR219.

In this study the ITS-rDNA region of two strains of Malaysian isolates of* Rhizoctonia solani* (1802/KB and 1801/UPM) recovered from two different local rice fields were sequenced to determine genetic diversity between the isolates. Following molecular characterization, these isolates were used in screening twenty varieties of local as well as international rice varieties that have been reported to show resistance against various rice diseases using the mycelial plug technique. The effectiveness of these techniques for screening experiments was tested. Two scoring techniques were also compared to verify techniques that truly represented the resistance of the varietal analysis. Varieties identified with some level of resistance or tolerance to* Rhizoctonia solani* isolates used in this study will be selected for use as donors in our breeding program.

## 2. Materials and Methods

### 2.1. Preparation of* Rhizoctonia solani* Culture and Their Maintenance

Two strains of* R. solani* isolates (strain 1801/UPM (accession number: KF312464) and strain 1802/KB (accession number: KF312465)) were collected and maintained on potato dextrose agar (PDA) at −80°C for long-term storage. Short-term cultures for immediate use were maintained at 4°C. These isolates were obtained from diseased rice tissues from paddy fields in Malaysia.

### 2.2. Molecular Characterization of Malaysian Isolates of* Rhizoctonia solani* DNA Extraction

Fresh* R. solani *cultures were used for subculturing in solid and liquid media. A 5 mm plug of mycelium was obtained from PDA plates and inoculated aseptically into potato dextrose broth (PDB). These cultures were maintained at 28°C for seven (7) days with agitation at 150 rpm [33]. The mycelial mat was harvested by funnel filtration, dried, and finely macerated in liquid nitrogen using micropestle. The macerated tissue was then used as source for DNA extraction. Genomic DNA of 1801/UPM and 1802/KB was extracted using DNeasy Plant Mini Kit (Qiagen, USA).

### 2.3. Amplification of rDNA-ITS Region

Internal transcribed spacer 1 (ITS1) and ITS2 regions, including the ribosomal 5.8S RNA gene, were amplified as one amplicon using specific primer pairs (Table 1) [34]. The PCR amplification reaction mixtures (25 *μ*L) contained 75 mm Tris-HCl (pH 8.8 at 25°C), 20 mm (NH_4_)_2_SO_4_, 1.5 mm MgCl_2_, 200 *μ*m dNTPs, 20 *μ*g mL^−1^ bovine serum albumin, 5 pmol of each primer, 2 *μ*L of template, and 1.25 units of* Taq* polymerase (MBI Fermentas, USA) [34]. Amplification was performed in a thermal cycler (Mastercycler; Eppendorf) using the following program: initial denaturation at 94°C for 5 min, followed by 34 cycles of denaturing at 94°C for 30 s, annealing at 58°C for 30 s and extension at 72°C for 1 min, and a final extension at 72°C for 10 min. As for 1801/UPM strain, the PCR mixture prepared was amplified using 4 min at 94°C, followed by 35 cycles of 1 min at 94°C, 1 min at 65°C, and 2 min at 72°C. The reaction was completed with a 5 min extension step.

### 2.4. DNA Sequencing

The PCR product obtained with ITS1/ITS4 pair for the isolates 1801/UPM and 1802/KB was purified via QIAquick PCR purification kit (Qiagen, USA) and was sequenced via Applied Biosystems 3730xl DNA Analyzer (USA). The forward and reverse sequences obtained were assembled using the Bioedit program (http://www.mbio.ncsu.edu/bioedit/bioedit.html).

### 2.5. ITS-rDNA Nucleotide Sequence Comparisons

The DNA sequence alignment of the isolates was generated via Multalin in order to observe any sequence variations. Identification to anastomosis groups was conducted based on comparison of 5.8S and ITS2 rDNA sequence similarity of more than 96% with other strain sequences of* R. solani* in the GenBank database [8] representing different anastomosis groups. The sequence data obtained from the sequencing process above was served as the query sequence and compared against all* Rhizoctonia* sp sequences using BLAST search available at http://www.ncbi.nlm.nih.gov. Additional reference sequences of 43 isolates from known AG groups worldwide were retrieved from GenBank database.

Multiple sequence alignments for homology search and phylogenetic analysis of downloaded sequences were performed using MEGA5.2 software (http://www.megasoftware.net/). In the Maximum Likelihood method for evaluating the fit of substitution models to the data, MEGA 5.2 automatically inferred the evolutionary tree by the Neighbor-Joining (NJ) algorithm using a matrix of pairwise distances estimated under the Tamura and Nei (1993) model for nucleotide sequences [2, 16, 35, 36] although there were five other nested models for DNA sequence analysis (Hasegawa-Kishino-Yano, Tamura three-parameter, Kimura two-parameter, Tajima-Nei, and Jukes-Cantor). The automatic option was used as trees are rarely known* a priori*.

A total of 68 sequences were used in the Maximum Likelihood analysis. The bootstrap consensus tree inferred from 1000 replicates [37] is taken to represent the evolutionary history of the taxa analyzed [37]. Branches corresponding to partitions reproduced in less than 50% bootstrap replicates are collapsed. Initial tree(s) for the heuristic search were obtained automatically by applying Neighbor-Join and BioNJ algorithms to a matrix of pairwise distances estimated using the Maximum Composite Likelihood (MCL) approach and then selecting the topology with superior log likelihood value. Codon positions included were 1st + 2nd + 3rd + Noncoding. All positions containing gaps and missing data were eliminated. There were a total of 539 positions in the final dataset.

The Maximum Parsimony tree was obtained using the Subtree-Pruning-Regrafting (SPR) algorithm (page 126 in [21]) with search level 1 in which the initial trees were obtained by the random addition of sequences (10 replicates). The analysis involved 45 nucleotide sequences inclusive of our two sequences. All positions containing gaps and missing data were eliminated. There were a total of 351 positions in the final dataset. The bootstrap consensus tree inferred from 1000 replicates is taken to represent the evolutionary history of the taxa analyzed [37]. The percentage of replicate trees in which the associated taxa clustered together in the bootstrap test (1000 replicates) is shown in the branches [37].

### 2.6. Screening for Sheath Blight Disease Paddy Cultivation

Twenty rice varieties, four local (MR219, MAHSURI, UKMRC2, and UKMRC9), and sixteen (IR8, IR20, TETEP, Teqing, IR24, IR36, IR64, Jasmine 85, C4-113, ADT 36, ADT 38, ADT 39, IR39-14, IR50, and TOX 2104-2-1) international varieties were selected for this study. Dormancy of these seeds was broken at 40°C for two days. The seeds were then germinated at 28°C with 16 hours day 8 hours night cycle in white light. The environment was kept aseptic to ensure that the seedlings were disease- and contaminant-free before transplantation into pots. Five days later, the germinated seeds were transferred into pots containing loam soil that was added with fertilizers.

### 2.7. *Rhizoctonia solani* Inoculum Preparation

Both the* Rhizoctonia* isolates* R. solani* 1801/UPM and* R. solani* 1802/KB mycelia or sclerotia were subcultured onto potato dextrose agar (PDA) and grown at room temperature (22 to 24°C) under continuous light. These cultures were used to prepare mycelial plugs from 3-day-old cultures [26].

### 2.8. Method of Infection

Rice plants at late tillering stage (10 week old plants) were inoculated with* R. solani* by placing mycelial plugs beneath the leaf sheath. The inoculated sheath was covered immediately with aluminum foil. When typical lesions appeared at day 3, the aluminum foil was removed and the infected plants were left in a surrounding that was maintained at 80–100% humidity. Plants were grown in ~30–32°C under natural light in standard greenhouse conditions [26].

### 2.9. Screening* Rhizoctonia solani* on Rice Plants

Inoculated plants were observed for three weeks and the data obtained was analyzed. Symptoms were scored by taking measurements of the lesions in five of the rice plant tissues: first leaf, second leaf, third leaf, fourth leaf, and fifth leaf. Each part has its own value calculation. The affected stem area index takes 20%, the first and second leaf 10%, the third and fourth leaf 15%, and finally the fifth leaf area 30% of the maximum percentage of the index value. These calculations take into account the length of each side of the wound lesions and area of stem and leaf length. The percentage is then used with the scoring table for* Rhizoctonia solani* effect on rice developed by the International Rice Research Institute (IRRI) (Table 2).

The index of disease susceptibility was calculated as follows: susceptibility index = (5*n*_5_ + 4*n* + 3*n*_3_ + 2*n*_2_ + 1*n*_1_ + 0*n*_0_)/5*N* × 100, where *n* is the number of leaves in each degree (0 to 5) and *N* is the number of total leaves investigated in a culm [26]. All lesions from a culm were added together to give a total lesion length for each culm. Three replications were used for each cultivar. The analysis of variance of the susceptibility index and the lesion length caused by* R. solani* was performed using SAS (version 6.12) (http://www.sas.com). Separation of cultivar means for both susceptibility index and lesion length was performed using Duncan's multiple range tests.

## 3. Results

### 3.1. Symptoms Evaluation of Isolates

**Figure 1:**
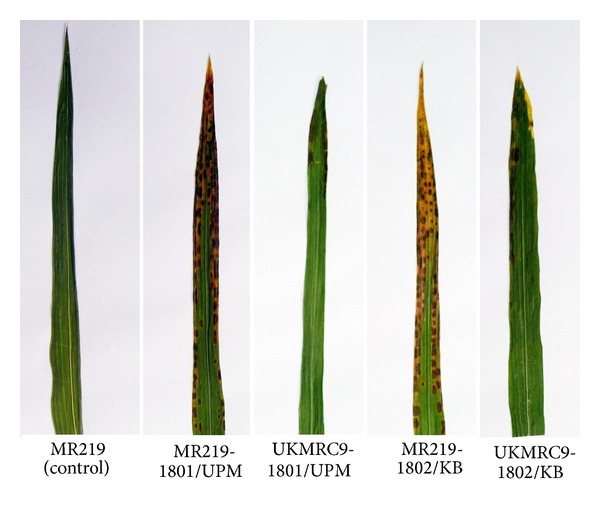
Disease symptoms of both isolates of* Rhizoctonia *spp on MR219 and UKMRC9. Disease symptoms were first exhibited in the sheath and then gradually progressed to infect the leave tissue as disease progressed.

The disease symptoms of both isolates of* Rhizoctonia *spp are displayed here on two Malaysian rice varieties, MR219 (developed by the Malaysia Agricultural Research and Development Institute), a widely cultivated rice variety and UKMRC9, an ABQTL derived rice variety produced through a cross generated between MR219 and* Oryza rufipogon* at Universiti Kebangsaan Malaysia.  Figure 1 displays the differences in disease symptoms shown by both varieties where the symptoms were more severe in MR219 compared to UKMRC9. More prominent blight symptoms were observed in strain 1802/KB compared to strain 1801/UPM.

### 3.2. ITS-rDNA Analysis of the* R. solani* Isolates

The 1801/UPM isolate was amplified using the primer pairs as in Table 1. Primer pairs ITS1/GMRS3 and ITS1/ITS4 successfully amplified the ITS region of this strain. An amplicon of ~550 bp was observed with primers ITS1/GMRS3 (Supplementary File, Figure 1(a) available online at http://dx.doi.org/10.1155/2014/434257) while primers ITS1/ITS4 produced amplicons of ~720 bp (Supplementary File, Figure 2(a)).

A similar approach was taken with strain 1802/KB where the same primer sets yielded ~550 bp amplicon (Supplementary File, Figure 1(b)) while primers ITS1/ITS4 produced a ~700 bp product (Supplementary File, Figure 2(b)). This is in line with the predicted size of amplicons expected with either primer pairs. However, the ITS1/GMRS3 primer pair was less consistent at producing results (Supplementary File, Figure 1). This could be due to the GMRS3 primer being derived from ITS2, where the ITS2 combination rendered no results in our study. The sequence derived from the use of ITS1/ITS4 was used in all further analyses.

### 3.3. Sequencing and Data Analysis

**Figure 2:**
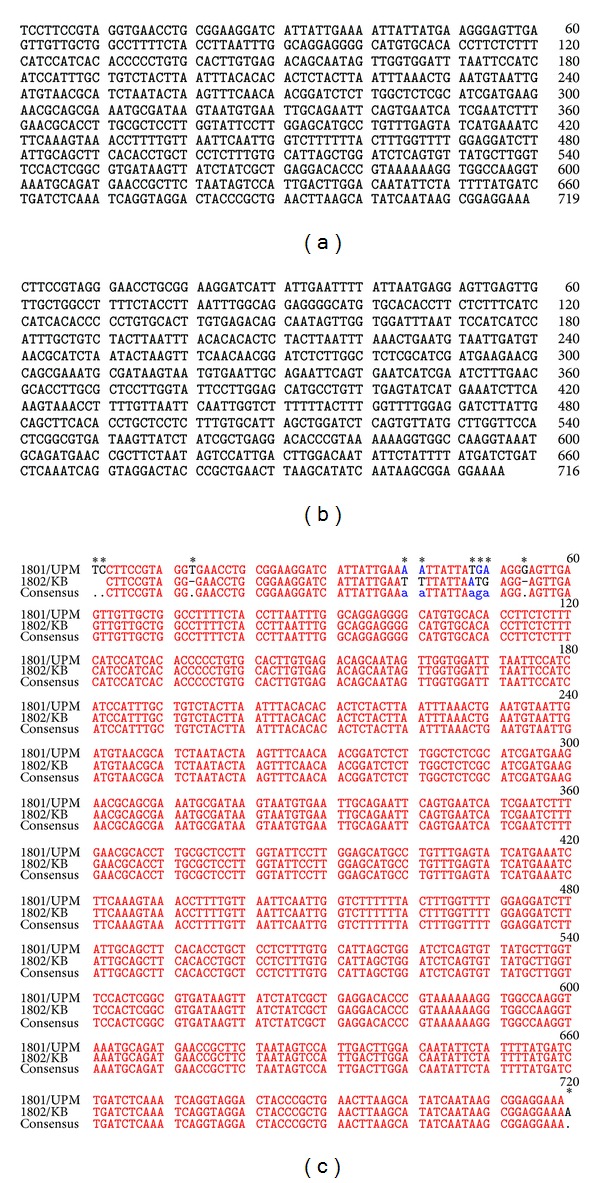
(a) The consensus ITS-rDNA sequence of strain 1801/UPM (719 bp). (b) The consensus ITS-rDNA sequence of strain 1802/KB (716 bp). Both consensus sequences were generated using the BioEdit program. (c) The consensus sequence of strain 1801/UPM and 1802/KB is different in the regions highlighted by an asterisk.

The consensus sequence of both isolates 1801/UPM and 1802/KB is presented in Figures 2(a) and 2(b). The consensus sequence for 1801/UPM was blasted in NCBI BLAST and generated 98 blast hits with the query showing maximum hit identity of 98-99%. The* Rhizoctonia* strains that are highly identical to 1801/UPM were mostly from China, Vietnam, India, and Japan and were obtained from paddy and maize tissues (Table 3(a)).

The sequences were compared and variations were observed between the two isolates. ITS sequences are known to result in variation within a single strain of* R. solani*. The sequence heterogeneity is believed to be caused by insertion or base substitution within the ITS region. This can be easily detected by PCR amplification as the ITS sequences of the amplified total rDNA population present.  Figure 2(c) shows the multiple insertion and substitution incidences that have occurred in the sequences of strain 1801/UPM and 1802/KB.

Similarly the consensus sequence of strain 1802/KB was also blasted against all nucleotide sequences in GenBank, and out of the 99 blast hit there were four subject sequences with maximum hit identity at 100% with the E-value of 0.0. The strain was highly identical to* Thanatephorus cucumeris* from China (FJ515881.1 and JF429710.1) and* Rhizoctonia solani *from India (JF701746.1) and the United States (JQ410052.1) (Table 3(b)).

**Figure 3:**
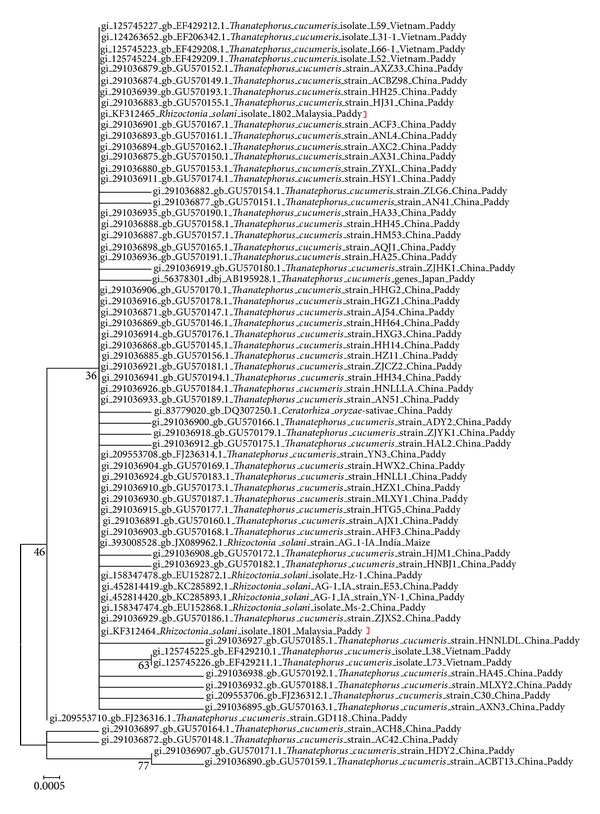
Maximum Likelihood analysis of ITS-rDNA nucleotide sequences of* Rhizoctonia solani* and* Thanatephorus *sp. Evolutionary analyses were conducted in MEGA 5.2 [36, 39]. The red brackets indicate the location of our isolates.

The results of the multiple alignment data generated were used to build the phylogenetic relationship between our isolates and those downloaded from NCBI. In this study a Maximum Likelihood analysis was conducted on 68 sequences of* Rhizoctonia solani *and teleomorph* Thanatephorus cucumeris *that were isolated fromrelated host (Figure 3). The MEGA 5.2 program was used to derive this tree where the trees were automatically generated using the Tamura-Nei model [38]. However in addition to the automatically generated tree the Juke Cantor Model was also used to generate a tree as this model too has been widely used in evolutionary studies of microorganisms. Both trees were compared and show the same distribution with slight variation in distance. However, we chose the Tamura-Nei model for its robustness and suitability in substitution analysis involving nucleotide sequences [16, 36, 39].

From the Maximum Likelihood tree constructed, both strains (1801/UPM and 1802/KB) were clustered under the same clade with other* Rhizoctonia* species and* Thanatephorus* species recovered from the Asian region with sequence identity that ranged from 98% to 100% (Figure 3). The downloaded ITS-rDNA sequence of* R. solani* and* T. cucumeris* recovered from these geographical regions are very much conserved. Besides being confined geographically to the Asian region, these strains were mostly derived from cereal crops such as paddy and maize. Even though our isolates have been clustered into different clades, both are still clustered together with isolates from the AG1-1A anastomosis group (strains cross-referenced with information inphylota.net). Their separation may be due to the difference due to geographical separation.

**Figure 4:**
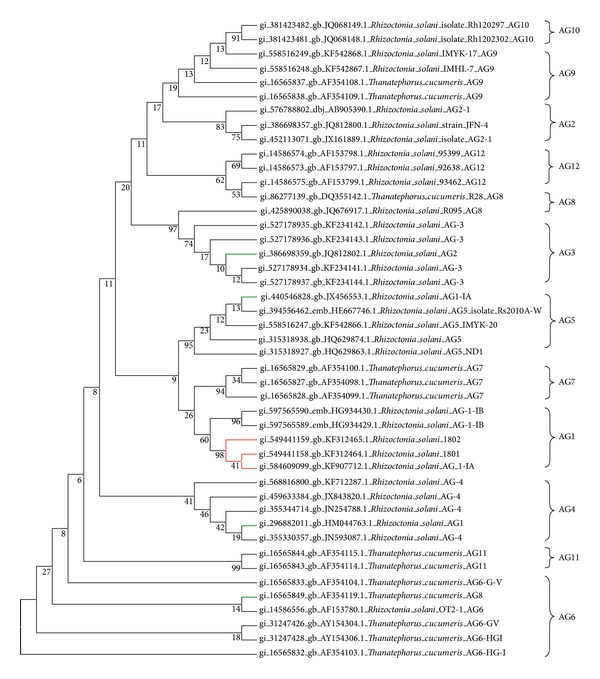
Maximum Parsimony Analysis of Taxa. The evolutionary history was inferred using the evolutionary analyses that were conducted in MEGA 5.2 [45]. The red lines show our field isolates are clustered together with* R. solani* AG 1-1A. The green lines indicate the strains that have segregated out of their AG location.

Additional 43 sequences representing 12 anastomosis groups (AG) with multiple sequences for each group were analyzed together with strains 1801/UPM and 1802/KB via Maximum Parsimony (Figure 4). Figure 4 shows that the separation may be due to the subgrouping where AGs generally form a distinct cluster. According to Ogoshi [40] and Sneh et al. [1], members of an AG often share certain pathogenic characteristics such as virulence and homogenous host-specific groups [41, 42], The details of the downloaded sequences were generally incomplete. From the analysis, the red box shows strong evidence that our* R. solani* isolates are most likely closely related to AG 1-1A compared to AG 1-1B. It has also been reported that AG 1-1A are likely to be pathogenic to rice [6–8]. It also appears that AG 1, AG 2, and AG 8 have been segregated out as there is branching of these anastomosis groups as observed in Figure 4. Figure 4 shows that AG 2 has evolved from the AG 3 and AG 8. It is known that these AGs are identified as being associated with the important cash and cereal crop disease. This result concurs with the findings of Pung et al. [43]. AG 8 has been clustered within the AG 6. The NCBI information of submitted sequences show that these AGs originated from the same country. Meanwhile, the AG 1-1A (gb∣44054688) shows some relatedness with AG 4 and AG 5 where these AGs infect cereal crops. Due to the host specificity of AGs it is likely that crops that are closely related like cereals may have related AG groupings. The subgroups of AGs may also cause different disease symptoms and be more dominant in certain plant species than others. This also could very well lead them to be clustered into different clades and a more widespread collection could be used to reveal deeper variations. It is therefore important to determine which AG is present in the field in order to manage the diseases properly [4].

### 3.4. Screening for Sheath Blight Disease

As the main objective for this research is to identify rice varieties that exhibit resistance or moderate resistance to* R. solani*, therefore it is important to select a quick, efficient, and consistent method for inoculation and screening for sheath blight in rice. In addition due to the fluctuation in temperatures (34–42°C) and humidity (80–100%) levels within the greenhouses, it is essential to select a method that would be reproducible amidst such variation in the environment. In this study, the method described by Park et al. [26] was used as it provides uniformity in inoculum size as mycelium plugs were used on all plants and 100% infection rate was achieved by covering the inoculated sheaths with aluminum foil. The foil retained the saturated humidity necessary for uniform and consistent infections which technically reduced the variation in disease development. The combination provided the reproducible infection necessary for performing the disease assays. The rapid and uniform infection of host tissues in this assay is critical for quantitative analysis of sheath blight resistance at molecular, biochemical, and genetical levels.

Tables 4 and 5 show the correlation between the average total lesion and severity index by strains 1801/UPM and 1802/KB. According to the results in both tables, the average total lesion produced by* Rhizoctonia solani* 1801/UPM and 1802/KB are positively correlated with a significance of 0.000 and may influence disease severity index [*r*(18) = 0.998  *P* < 0.001 for strain 1801 and *r*(18) = 0.996  *P* < 0.001 for strain 1802]. Tables 6 and 7 provide the disease extent, scoring scale, and type of resistance/susceptibility exhibited by the 20 cultivars screened in this experiment for strains 1801/UPM and 1802/KB, respectively.  Table 6 shows that variety TETEP, Jasmine 85, and Teqing were resistant to strain 1801/UPM while UKMRC9, IR8, MAHSURI, ADT 39, and TOX 2104-2-1 were moderately resistant to this strain. The most susceptible variety against 1801/UPM is IR20. When strain 1802/KB was used against the same varieties, only three were moderately resistant, that is, Teqing, TETEP, and Jasmine 85 (Table 7). The previously moderately resistant varieties were now either moderately susceptible or susceptible to this strain. This therefore indicates that strain 1802/KB is more virulent based on the disease severity values determined in this study.

**Figure 5:**
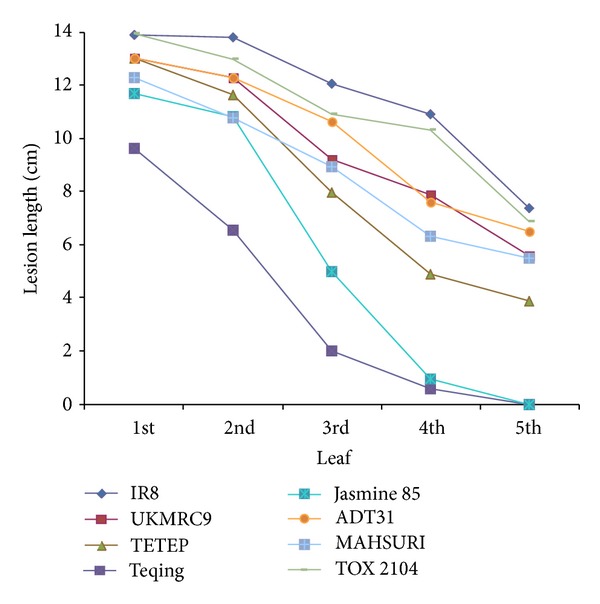
The lesion lengths observed in the top eight cultivars in this study. Lesion length taken in cm when treated with* R. solani* strain 1802/KB.

**Figure 6:**
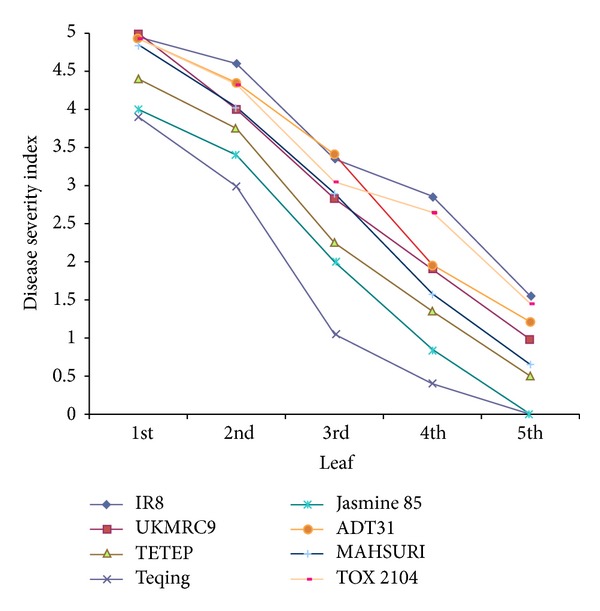
Disease severity of sheath blight in the top eight cultivars used in this study. Plants were treated with* R. solani* strain 1802/KB.

The two disease severity rating systems are shown graphically for both lesion length (Figure 5) and disease susceptibility index (Figure 6) across each leaf position above the inoculation point for the top eight cultivars based on the scores from Tables 6 and 7. The effect of cultivar was significant for both lesion length and susceptibility index (*P* < 0.001). Although both methods of measuring severity of sheath blight produced the same general trends (Figures 5 and 6), the susceptibility index was clearer and more consistent across all leaf positions within and across cultivars (Figures 5 and 6).

Disease severity ratings using lesion length and susceptibility index were highly correlated (Tables 4 and 5), with notable differences. One such difference was the lesion length for TETEP which was larger than most other cultivars (Figure 5) but showed a disease susceptibility index that was lower than other cultivars (Figure 6) [1, 35]. Similar results were also observed in Jasmine 85 where the lesion lengths were long but its susceptibility index was low.

Disease severity decreased as distance from the source of inoculum increased. When the infected cultivars were ranked, using either total lesion length or disease susceptibility index (Table 8), the susceptibility index rankings were more consistent with the published reports than the lesion length measurements [39, 44]. The susceptibility index also provided a clean differentiation of cultivars based on mean separation (Table 8 and Figure 6).

## 4. Discussion

### 4.1. Symptoms Evaluation of Isolates

The strains showed different disease severity on MR219 (susceptible rice variety) and UKMRC9 (moderately resistant variety) where strain 1802/KB was more virulent. When both strains were tested on the twenty rice varieties, similar results were obtained where the symptoms were more severe in 1802/KB inoculated samples compared to strain 1801/UPM.

### 4.2. ITS-rDNA Analysis of the* R. solani* Isolates

Analysis of nuclear rDNA genes, particularly in the internal transcribed spacer (ITS) regions, is good target for determining interspecies variations and phylogenetic analysis of fungal species [5, 22]. The rDNA region contains both variable and conserved regions allowing the comparison and discrimination of organisms at different taxonomic level. The internal transcribed spacers (ITS 1 and ITS 2) of 5.8S rRNA gene evolve relatively quickly and can be useful in determining interspecies variations and phylogenetic evolution. Fang et al. [46] have demonstrated previously that grouping of ITS sequences of* Rhizoctonia* isolates supports the AG grouping of* Rhizoctonia* isolates based on classical hyphal anastomosis reactions. This study confirms that molecular analysis based on the ITS sequences is appropriate for evaluating genetic diversity and characterizing potential AG groups of* Rhizoctonia* isolates. Each AG seems to be genetically independent from all others [26, 47] as seen in Figure 4 where AG 1, AG 2, AG 3, AG 4, AG 5, AG 6, AG 7, AG 8, AG 9, AG 10, and AG 11 were clustered into different subgroups.

In determining the phylactic lineages of strain 1801/UPM and 1802/KB, several primer sets were used to amplify the 5.8 rRNA gene regions. The ITS1 and ITS4 primers were used in the amplification of* R. solani *strain 1801/UPM and 1802/KB. The amplified product size is within the range reported by Johansson et al. [32] and Pannecoucque et al. [31] of between 600 and 750 bp. According to Pascual et al. [30] and Pannecoucque et al. [31], the total length of the ITS regions is expected to vary according to the primer pairs used.

When the sequences of strains 1801/UPM and 1802/KB were aligned, incidences of insertions and substitution were noted within the sequences. As* R. solani* is known to be multinucleate [1], the heterogeneity could also be due to chromosome variation present in different nuclei within the same strain. It is also possible that the insertion or substitution occurred in some of the approximately 200 copies [22] of the ITS sequence on the same chromosome, or that the same nucleus has different rDNAs on different chromosomes.

### 4.3. Phylogenetic Analysis

Based on the substitution incidences observed in our sequences, the Tamura and Nei (1993) model seems biologically most plausible as it uses Gamma distribution that results in continuous distribution in which every site may have a different rate change as opposed to fraction site changes at one rate or invariably especially for rapidly evolving groups of organisms such as microbes [16, 19]. The phylogenetic analysis of our two isolates against the 66 sequences downloaded from NCBI showed that our strains were highly identical (98–100%) to other regionally related isolates (Asia) and certain* R. solani* and* Thanatephorus cucumeris* from United States (100%), United Kingdom (99%), Switzerland (99%), and Colombia (99%). Linde et al. [48] reported that the gene flow of* R. solani* genotype is usually within a distance of 280 metres. From our phylactic studies however we find that the gene flow may have gone beyond this restriction on distance [48] where high levels of identity in isolates were observed at sequence level for isolates within the region and beyond. We believe that the gene flow of the isolates may have been assisted by trade relations within the region [7]. Since Malaysia imports and exports certain grains to and from these countries, it is possible that the pathogen was present in these sources and survived for some time until they found favourable conditions and regenerated and thus contributed to the gene flow of* R. solani* genotypes [7].

Besides the gene flow by* R. solani* genotypes, host-relatedness may also be another contributing factor (rice and maize). Butler [49] stated that AGs appear to be plant host specific. Therefore, it becomes easier for the divergence of populations through selection of specific host plants. The extent of differentiation and specialization of* R. solani* AG populations is probably dependent on the degree of relatedness of their hosts of origin as seen from our results where our isolates were closely related to AG 1-1A that was reported to be the anastomosis group involved in rice infections [1]. The divergence can also be generated through the selection imposed by different environmental factors such as the cropping system [50] or divergence through nondescendent.

### 4.4. Screening for Sheath Blight Disease

A comparison of two methods for rating the disease severity was made to determine which method produced the most accurate disease severity data scoring among twenty cultivars [17, 51]. The first method measured total lesion length and the second method used the disease susceptibility index.

From the data collected we were able to conclude that the susceptibility index is more consistent and a better way of evaluating the disease severity in rice. One contributing factor to the variation observed between lesion length and disease severity index lies in the plant architecture. Plant architecture of the twenty cultivars used in this study varied in height, leaf width, and length. As a result, the disease susceptibility index is a more accurate measure of disease severity as it is not skewed by cultivar differences in plant architecture as are direct measurements of lesion length. One weakness seen in lesion length measurement is that it does not take into account the width of the lesion. If a lesion is long but narrow, it may not result in severe disease effect as solutes may still move across the tissue while a short wide lesion may result in obstruction of solute flow and therefore result in more severe disease symptoms.

Researchers have also discovered that the same cultivar can vary in disease severity index due to environmental effects. This is why a screening method that is stable and reproducible is emphasized. It is important to establish a method that is reproducible in any research facility that may even have limited resources in form of facilities and capabilities.

The selection of resistant cultivars is the most economically and environmentally beneficial way of reducing losses to sheath blight in rice. As it is important for us to use all our field isolates in screening for resistance, we isolated and conducted molecular characterization on our field isolates before selecting two distinct strains to use in the screening experiments. Cultural control methods [6] are insufficient and the use of fungicides [52] may not be economically or environmentally sustainable. Transformation of rice cultivars with defense genes has provided only partial resistance [20]. To achieve selection of candidate cultivars for breeding programs, a uniform and effective infection and accurate evaluation method is required for detailed genetic, molecular, biochemical, and functional genomics analyses for measuring quantitative differences in sheath blight resistance among rice breeding lines, mutants, and transgenic plants. Most researchers have used methods that have been reported in Jia et al. [53]. However here we have used a simple inoculation method that produced stable, consistent, and reproducible infections in inoculated tissues. The lesion length and susceptibility index values were observed and the results weighed the susceptibility values as more consistent. The overall outcome of this research are the molecular characterization of our* R. solani* field isolates, the identification of Teqing, Tetep and Jasmine 85 as possible donor candidates for our breeding program, the consistency of fungal plug inoculation system for use in the screening experiments, and the selection of the susceptibility index as a more valid measure of disease severity compared to lesion length in disease scoring. These findings will provide a solid basis for our future breeding and screening activities at the institution.

## Supplementary Material

The 1801/UPM was amplified using the primer pairs as in Table 1. Primer pairs ITS1/GMRS3 and ITS1/ITS4 successfully amplified the ITS region of this strain. An amplicon of ~550 bp was observed with primers ITS1/GMRS3 (Supplementary File, Figure 1A and 1B) while primers ITS1/ITS4 produced amplicons of ~720 bp (Supplementary File, Figure 2A and 2B).

## Figures and Tables

**Table 1:** Primer sequences specific for *Rhizoctonia* species.

Set	Primer	Sequence (5′-3′)	Direction^a^	Estimated product size (bp)
1^b^	ITS 1	TCC GTA GGT GAA CCT GCG G	F	600–750
	ITS 4	TCC TCC GTT ATT GAT ATG C	R	

2^c^	ITS 1	TCC GTA GGT GAA CCT GCG G	F	550
	GMRS-3	AGT GGA ACC AAG CAT AAC ACT	R	

^a^F: forward primer; R: reverse primer.

^b^Pascual et al. [30]; Pannecoucque et al. [31].

^c^Johansson et al. [32].

**Table 2:** IRRI scoring schedule for sheath blight disease (*Rhizoctonia solani*) (0–9 scale).

Types	Symptoms shown
HR	No changes
R	Lesions limited to below 1/4 of the leaf
MR	Lesions limited to below 1/2 of the leaves
MS	Lesions present in more than 1/2 of the leaves
S	Lesions present in more than 1/4 of the leaf surface. Severe infection in upper leaves (2 branches of withered leaves)
HS	Lesions reach the tiller. Severe infection on all leaves and some plants were killed.

Description: HS: highly susceptible; S: susceptible; MS: moderately susceptible; MR: moderately resistant; R: resistant; HR: highly resistant.

**(a):** 

ID/accession	Organisms	Identity (%)	Max score
gi∣291036941∣gb∣GU570194.1∣	*Thanatephorus cucumeris* strain HH34	99.3	1290
gi∣291036930∣gb∣GU570187.1∣	*Thanatephorus cucumeris* strain MLXY1	99.3	1290
gi∣291036929∣gb∣GU570186.1∣	*Thanatephorus cucumeris* strain ZJXS2	99.3	1290
gi∣291036924∣gb∣GU570183.1∣	*Thanatephorus cucumeris* strain HNLL1	99.3	1290
gi∣291036921∣gb∣GU570181.1∣	*Thanatephorus cucumeris* strain ZJCZ2	99.3	1290
gi∣291036916∣gb∣GU570178.1∣	*Thanatephorus cucumeris* strain HGZ1	99.3	1290
gi∣291036914∣gb∣GU570176.1∣	*Thanatephorus cucumeris* strain HXG3	99.3	1290
gi∣291036910∣gb∣GU570173.1∣	*Thanatephorus cucumeris* strain HZX1	99.3	1290
gi∣291036906∣gb∣GU570170.1∣	*Thanatephorus cucumeris* strain HHG2	99.3	1290
gi∣291036904∣gb∣GU570169.1∣	*Thanatephorus cucumeris* strain HWX2	99.3	1290
gi∣291036885∣gb∣GU570156.1∣	*Thanatephorus cucumeris* strain HZ11	99.3	1290
gi∣291036871∣gb∣GU570147.1∣	*Thanatephorus cucumeris* strain AJ54	99.3	1290
gi∣291036869∣gb∣GU570146.1∣	*Thanatephorus cucumeris* strain HH64	99.3	1290
gi∣291036868∣gb∣GU570145.1∣	*Thanatephorus cucumeris* strain HH14	99.3	1290
gi∣209553708∣gb∣FJ236314.1∣	*Thanatephorus cucumeris* strain YN3	99.3	1290
gi∣307948734∣gb∣HQ270162.1∣	*Thanatephorus cucumeris* isolate YR-33	99.3	1288
gi∣307948730∣gb∣HQ185376.1∣	*Thanatephorus cucumeris* isolate YR-167	99.3	1288
gi∣307948724∣gb∣HQ185370.1∣	*Thanatephorus cucumeris* isolate YR-52	99.3	1288
gi∣291036901∣gb∣GU570167.1∣	*Thanatephorus cucumeris* strain ACF3	99.3	1288
gi∣291036898∣gb∣GU570165.1∣	*Thanatephorus cucumeris* strain AQJ1	99.3	1288
gi∣291036894∣gb∣GU570162.1∣	*Thanatephorus cucumeris* strain AXC2	99.3	1288
gi∣291036893∣gb∣GU570161.1∣	*Thanatephorus cucumeris* strain ANL4	99.3	1288
gi∣291036887∣gb∣GU570157.1∣	*Thanatephorus cucumeris* strain HM53	99.3	1288
gi∣216963574∣gb∣FJ440186.1∣	*Thanatephorus cucumeris* isolate YWK-3	99.3	1288
gi∣56378301∣dbj∣AB195928.1∣	*Thanatephorus cucumeris* genes	99.16	1288
gi∣291036936∣gb∣GU570191.1∣	*Thanatephorus cucumeris* strain HA25	99.16	1286
gi∣291036933∣gb∣GU570189.1∣	*Thanatephorus cucumeris* strain AN51	99.16	1286
gi∣291036926∣gb∣GU570184.1∣	*Thanatephorus cucumeris* strain HNLLLA	99.16	1286
gi∣291036883∣gb∣GU570155.1∣	*Thanatephorus cucumeris* strain HJ31	99.3	1286
gi∣291036918∣gb∣GU570179.1∣	*Thanatephorus cucumeris* strain ZJYK1	99.16	1284
gi∣291036915∣gb∣GU570177.1∣	*Thanatephorus cucumeris* strain HTG5	99.16	1284
gi∣291036912∣gb∣GU570175.1∣	*Thanatephorus cucumeris* strain HAL2	99.16	1284
gi∣291036908∣gb∣GU570172.1∣	*Thanatephorus cucumeris* strain HJM1	99.16	1284
gi∣291036900∣gb∣GU570166.1∣	*Thanatephorus cucumeris* strain ADY2	99.16	1284
gi∣209553710∣gb∣FJ236316.1∣	*Thanatephorus cucumeris* strain GD118	99.16	1284
gi∣291036939∣gb∣GU570193.1∣	*Thanatephorus cucumeris* strain HH25	99.16	1282
gi∣83779020∣gb∣DQ307250.1∣	*Ceratorhiza oryzae-sativae *	99.16	1282
gi∣326633412∣gb∣JF701760.1∣	*Rhizoctonia solani* isolate RPBU5	98.75	1279
gi∣291036927∣gb∣GU570185.1∣	*Thanatephorus cucumeris* strain HNNLDL	99.02	1279
gi∣291036888∣gb∣GU570158.1∣	*Thanatephorus cucumeris* strain HH45	99.02	1279
gi∣291036938∣gb∣GU570192.1∣	*Thanatephorus cucumeris* strain HA45	99.02	1277
gi∣291036932∣gb∣GU570188.1∣	*Thanatephorus cucumeris* strain MLXY2	99.02	1277
gi∣216963633∣gb∣FJ440210.1∣	*Thanatephorus cucumeris* isolate YWK-196	99.02	1277
gi∣291036923∣gb∣GU570182.1∣	*Thanatephorus cucumeris* strain HNBJ1	98.88	1273
gi∣326633386∣gb∣JF701734.1∣	*Rhizoctonia solani* isolate RHRW27	98.75	1271
gi∣291036874∣gb∣GU570149.1∣	*Thanatephorus cucumeris* strain ACBZ98	98.88	1271
gi∣216963603∣gb∣FJ440192.1∣	*Thanatephorus cucumeris* isolate YWK-62	99.29	1271
gi∣432134622∣gb∣JX913816.1∣	*Rhizoctonia solani* isolate Y1075	98.75	1269
gi∣326633400∣gb∣JF701748.1∣	*Rhizoctonia solani* isolate RMPM13	98.61	1269
gi∣291036903∣gb∣GU570168.1∣	*Thanatephorus cucumeris* strain AHF3	98.88	1269
gi∣291036897∣gb∣GU570164.1∣	*Thanatephorus cucumeris* strain ACH8	98.74	1269
gi∣291036891∣gb∣GU570160.1∣	*Thanatephorus cucumeris* strain AJX1	98.88	1269
gi∣125745230∣gb∣EF429215.1∣	*Thanatephorus cucumeris* isolate LB71	99.29	1267
gi∣125745229∣gb∣EF429214.1∣	*Thanatephorus cucumeris* isolate DP38	99.29	1267
gi∣125745227∣gb∣EF429212.1∣	*Thanatephorus cucumeris* isolate L59	99.29	1267
gi∣326633407∣gb∣JF701755.1∣	*Rhizoctonia solani* isolate RMHG24	98.48	1266
gi∣291036935∣gb∣GU570190.1∣	*Thanatephorus cucumeris* strain HA33	98.74	1266
gi∣291036911∣gb∣GU570174.1∣	*Thanatephorus cucumeris* strain HSY1	98.74	1266
gi∣291036880∣gb∣GU570153.1∣	*Thanatephorus cucumeris* strain ZYXL	98.74	1266
gi∣291036875∣gb∣GU570150.1∣	*Thanatephorus cucumeris* strain AX31	98.74	1266
gi∣291036872∣gb∣GU570148.1∣	*Thanatephorus cucumeris* strain AC42	98.61	1266
gi∣216963623∣gb∣FJ440207.1∣	*Thanatephorus cucumeris* isolate YWK-169	98.74	1266
gi∣216963617∣gb∣FJ440201.1∣	*Thanatephorus cucumeris* isolate YWK-120	99.29	1266
gi∣125745231∣gb∣EF429216.1∣	*Thanatephorus cucumeris* isolate RM61	99.29	1266
gi∣125745224∣gb∣EF429209.1∣	*Thanatephorus cucumeris* isolate L52	99.29	1266
gi∣124263651∣gb∣EF206341.1∣	*Thanatephorus cucumeris* isolate BV50-1	99.29	1266
gi∣307948721∣gb∣HQ185367.1∣	*Thanatephorus cucumeris* isolate YR-23	98.74	1264
gi∣291036879∣gb∣GU570152.1∣	*Thanatephorus cucumeris* strain AXZ33	98.74	1264
gi∣125745223∣gb∣EF429208.1∣	*Thanatephorus cucumeris* isolate L66-1	99.15	1264
gi∣125745228∣gb∣EF429213.1∣	*Thanatephorus cucumeris* isolate CLV72-2	99.29	1262
gi∣125745226∣gb∣EF429211.1∣	*Thanatephorus cucumeris* isolate L73	99.15	1262
gi∣124263652∣gb∣EF206342.1∣	*Thanatephorus cucumeris* isolate L31-1	99.15	1262
gi∣326633424∣gb∣JF701772.1∣	*Rhizoctonia solani* isolate RUPM42	98.87	1260
gi∣291036919∣gb∣GU570180.1∣	*Thanatephorus cucumeris* strain ZJHK1	98.6	1260
gi∣291036882∣gb∣GU570154.1∣	*Thanatephorus cucumeris* strain ZLG6	98.6	1260
gi∣125745225∣gb∣EF429210.1∣	*Thanatephorus cucumeris* isolate L38	99.15	1260
gi∣291036877∣gb∣GU570151.1∣	*Thanatephorus cucumeris* strain AN41	98.6	1258
gi∣122892471∣gb∣EF187916.1∣	*Thanatephorus cucumeris* strain DSM	98.6	1258
gi∣209553706∣gb∣FJ236312.1∣	*Thanatephorus cucumeris* strain C30	98.47	1256
gi∣291036895∣gb∣GU570163.1∣	*Thanatephorus cucumeris* strain AXN3	98.46	1254
gi∣291036907∣gb∣GU570171.1∣	*Thanatephorus cucumeris* strain HDY2	98.32	1247
gi∣219972597∣gb∣FJ515881.1∣	*Thanatephorus cucumeris* isolate CHR08-01	99.28	1247
gi∣216963639∣gb∣FJ440211.1∣	*Thanatephorus cucumeris* isolate YWK-208	98.58	1242
gi∣326633398∣gb∣JF701746.1∣	*Rhizoctonia solani* isolate RMPM9	99.27	1240
gi∣326633427∣gb∣JF701775.1∣	*Rhizoctonia solani *isolate RUPU82	98.18	1238
gi∣534289541∣gb∣KF053536.1∣	*Rhizoctonia solani *isolate GDHY38	99.7	1236
gi∣534289540∣gb∣KF053535.1∣	*Rhizoctonia solan*i isolate GDHZ12	99.7	1236
gi∣326633385∣gb∣JF701733.1∣	*Rhizoctonia solani* isolate RHRW16	98.99	1234
gi∣326633425∣gb∣JF701773.1∣	*Rhizoctonia solani* isolate RUPM83	97.64	1230
gi∣291036890∣gb∣GU570159.1∣	*Thanatephorus cucumeris* strain ACBT13	97.65	1229
gi∣219972641∣gb∣FJ515885.1∣	*Thanatephorus cucumeris* isolate CHR08-14	98.55	1219
gi∣534289539∣gb∣KF053534.1∣	*Rhizoctonia solani* isolate JXWD5	99.26	1218
gi∣333034239∣gb∣JF429710.1∣	*Thanatephorus cucumeris* strain JS-1	99.26	1218
gi∣158347474∣gb∣EU152868.1∣	*Rhizoctonia solani *isolate Ms-2	99.26	1218
gi∣452814420∣gb∣KC285893.1∣	*Rhizoctonia solani* AG-1 IA strain YN-1	99.7	1216
gi∣385215144∣gb∣JQ410052.1∣	*Rhizoctonia solani* isolate 0465	99.26	1216
gi∣42475513∣dbj∣AB122135.1∣	*Thanatephorus cucumeris* genes	99.11	1210
gi∣42475511∣dbj∣AB122133.1∣	*Thanatephorus cucumeris* genes	99.11	1210

**(b):** 

ID/accession	Organisms	Identity (%)	Max Score
gi∣291036941∣gb∣GU570194.1∣	*Thanatephorus cucumeris* strain HH34	99.86	1310
gi∣291036930∣gb∣GU570187.1∣	*Thanatephorus cucumeris* strain MLXY1	99.86	1310
gi∣291036929∣gb∣GU570186.1∣	*Thanatephorus cucumeris* strain ZJXS2	99.86	1310
gi∣291036924∣gb∣GU570183.1∣	*Thanatephorus cucumeris* strain HNLL1	99.86	1310
gi∣291036921∣gb∣GU570181.1∣	*Thanatephorus cucumeris* strain ZJCZ2	99.86	1310
gi∣291036916∣gb∣GU570178.1∣	*Thanatephorus cucumeris* strain HGZ1	99.86	1310
gi∣291036914∣gb∣GU570176.1∣	*Thanatephorus cucumeris* strain HXG3	99.86	1310
gi∣291036910∣gb∣GU570173.1∣	*Thanatephorus cucumeris* strain HZX1	99.86	1310
gi∣291036906∣gb∣GU570170.1∣	*Thanatephorus cucumeris* strain HHG2	99.86	1310
gi∣291036904∣gb∣GU570169.1∣	*Thanatephorus cucumeris* strain HWX2	99.86	1310
gi∣291036885∣gb∣GU570156.1∣	*Thanatephorus cucumeris* strain HZ11	99.86	1310
gi∣291036871∣gb∣GU570147.1∣	*Thanatephorus cucumeris* strain AJ54	99.86	1310
gi∣291036869∣gb∣GU570146.1∣	*Thanatephorus cucumeris* strain HH64	99.86	1310
gi∣291036868∣gb∣GU570145.1∣	*Thanatephorus cucumeris* strain HH14	99.86	1310
gi∣209553708∣gb∣FJ236314.1∣	*Thanatephorus cucumeris* strain YN3	99.86	1310
gi∣56378301∣dbj∣AB195928.1∣	*Thanatephorus cucumeris* genes	99.72	1310
gi∣307948734∣gb∣HQ270162.1∣	*Thanatephorus cucumeris* isolate YR-33	99.86	1308
gi∣307948730∣gb∣HQ185376.1∣	*Thanatephorus cucumeris* isolate YR-167	99.86	1308
gi∣307948724∣gb∣HQ185370.1∣	*Thanatephorus cucumeris* isolate YR-52	99.86	1308
gi∣291036901∣gb∣GU570167.1∣	*Thanatephorus cucumeris* strain ACF3	99.86	1308
gi∣291036898∣gb∣GU570165.1∣	*Thanatephorus cucumeris* strain AQJ1	99.86	1308
gi∣291036894∣gb∣GU570162.1∣	*Thanatephorus cucumeris* strain AXC2	99.86	1308
gi∣291036893∣gb∣GU570161.1∣	*Thanatephorus cucumeris* strain ANL4	99.86	1308
gi∣291036887∣gb∣GU570157.1∣	*Thanatephorus cucumeris* strain HM53	99.86	1308
gi∣216963574∣gb∣FJ440186.1∣	*Thanatephorus cucumeris* isolate YWK-3	99.86	1308
gi∣291036936∣gb∣GU570191.1∣	*Thanatephorus cucumeris* strain HA25	99.72	1306
gi∣291036933∣gb∣GU570189.1∣	*Thanatephorus cucumeris* strain AN51	99.72	1306
gi∣291036926∣gb∣GU570184.1∣	*Thanatephorus cucumeris* strain HNLLLA	99.72	1306
gi∣291036883∣gb∣GU570155.1∣	*Thanatephorus cucumeris* strain HJ31	99.86	1306
gi∣291036918∣gb∣GU570179.1∣	*Thanatephorus cucumeris* strain ZJYK1	99.72	1304
gi∣291036915∣gb∣GU570177.1∣	*Thanatephorus cucumeris* strain HTG5	99.72	1304
gi∣291036912∣gb∣GU570175.1∣	*Thanatephorus cucumeris* strain HAL2	99.72	1304
gi∣291036908∣gb∣GU570172.1∣	*Thanatephorus cucumeris* strain HJM1	99.72	1304
gi∣291036900∣gb∣GU570166.1∣	*Thanatephorus cucumeris* strain ADY2	99.72	1304
gi∣209553710∣gb∣FJ236316.1∣	*Thanatephorus cucumeris* strain GD118	99.72	1304
gi∣291036939∣gb∣GU570193.1∣	*Thanatephorus cucumeris* strain HH25	99.72	1303
gi∣83779020∣gb∣DQ307250.1∣	*Ceratorhiza oryzae-sativae *	99.72	1303
gi∣291036927∣gb∣GU570185.1∣	*Thanatephorus cucumeris* strain HNNLDL	99.58	1299
gi∣291036888∣gb∣GU570158.1∣	*Thanatephorus cucumeris* strain HH45	99.58	1299
gi∣291036938∣gb∣GU570192.1∣	*Thanatephorus cucumeris* strain HA45	99.58	1297
gi∣291036932∣gb∣GU570188.1∣	*Thanatephorus cucumeris* strain MLXY2	99.58	1297
gi∣216963633∣gb∣FJ440210.1∣	*Thanatephorus cucumeris* isolate YWK-196	99.58	1297
gi∣326633386∣gb∣JF701734.1∣	*Rhizoctonia solani* isolate RHRW27	99.3	1293
gi∣291036923∣gb∣GU570182.1∣	*Thanatephorus cucumeris* strain HNBJ1	99.44	1293
gi∣326633400∣gb∣JF701748.1∣	*Rhizoctonia solani* isolate RMPM13	99.17	1291
gi∣291036874∣gb∣GU570149.1∣	*Thanatephorus cucumeris* strain ACBZ98	99.44	1291
gi∣216963603∣gb∣FJ440192.1∣	*Thanatephorus cucumeris* isolate YWK-62	99.86	1291
gi∣549441158∣gb∣KF312464.1∣	*Rhizoctonia solani *isolate 1801	99.16	1290
gi∣291036903∣gb∣GU570168.1∣	*Thanatephorus cucumeris* strain AHF3	99.44	1290
gi∣291036897∣gb∣GU570164.1∣	*Thanatephorus cucumeris* strain ACH8	99.3	1290
gi∣291036891∣gb∣GU570160.1∣	*Thanatephorus cucumeris* strain AJX1	99.44	1290
gi∣125745230∣gb∣EF429215.1∣	*Thanatephorus cucumeris* isolate LB71	99.86	1288
gi∣125745229∣gb∣EF429214.1∣	*Thanatephorus cucumeris* isolate DP38	99.86	1288
gi∣125745227∣gb∣EF429212.1∣	*Thanatephorus cucumeris* isolate L59	99.86	1288
gi∣326633424∣gb∣JF701772.1∣	*Rhizoctonia solani* isolate RUPM42	99.58	1286
gi∣326633407∣gb∣JF701755.1∣	*Rhizoctonia solani* isolate RMHG24	99.03	1286
gi∣291036935∣gb∣GU570190.1∣	*Thanatephorus cucumeris* strain HA33	99.3	1286
gi∣291036911∣gb∣GU570174.1∣	*Thanatephorus cucumeris* strain HSY1	99.3	1286
gi∣291036880∣gb∣GU570153.1∣	*Thanatephorus cucumeris* strain ZYXL	99.3	1286
gi∣291036875∣gb∣GU570150.1∣	*Thanatephorus cucumeris* strain AX31	99.3	1286
gi∣291036872∣gb∣GU570148.1∣	*Thanatephorus cucumeris* strain AC42	99.16	1286
gi∣216963623∣gb∣FJ440207.1∣	*Thanatephorus cucumeris* isolate YWK-169	99.3	1286
gi∣216963617∣gb∣FJ440201.1∣	*Thanatephorus cucumeris* isolate YWK-120	99.86	1286
gi∣125745231∣gb∣EF429216.1∣	*Thanatephorus cucumeris* isolate RM61	99.86	1286
gi∣125745224∣gb∣EF429209.1∣	*Thanatephorus cucumeris* isolate L52	99.86	1286
gi∣124263651∣gb∣EF206341.1∣	*Thanatephorus cucumeris* isolate BV50-1	99.86	1286
gi∣307948721∣gb∣HQ185367.1∣	*Thanatephorus cucumeris* isolate YR-23	99.3	1284
gi∣291036879∣gb∣GU570152.1∣	*Thanatephorus cucumeris* strain AXZ33	99.3	1284
gi∣125745223∣gb∣EF429208.1∣	*Thanatephorus cucumeris* isolate L66-1	99.72	1284
gi∣326633412∣gb∣JF701760.1∣	*Rhizoctonia solani *isolate RPBU5	98.89	1282
gi∣125745228∣gb∣EF429213.1∣	*Thanatephorus cucumeris* isolate CLV72-2	99.86	1282
gi∣125745226∣gb∣EF429211.1∣	*Thanatephorus cucumeris* isolate L73	99.71	1282
gi∣124263652∣gb∣EF206342.1∣	*Thanatephorus cucumeris* isolate L31-1	99.71	1282
gi∣291036919∣gb∣GU570180.1∣	*Thanatephorus cucumeris* strain ZJHK1	99.16	1280
gi∣291036882∣gb∣GU570154.1∣	*Thanatephorus cucumeris* strain ZLG6	99.16	1280
gi∣125745225∣gb∣EF429210.1∣	*Thanatephorus cucumeris* isolate L38	99.71	1280
gi∣432134622∣gb∣JX913816.1∣	*Rhizoctonia solani* isolate Y1075	99.02	1279
gi∣291036877∣gb∣GU570151.1∣	*Thanatephorus cucumeris* strain AN41	99.16	1279
gi∣122892471∣gb∣EF187916.1∣	*Thanatephorus cucumeris* strain DSM 843	99.16	1279
gi∣209553706∣gb∣FJ236312.1∣	*Thanatephorus cucumeris* strain C30	99.02	1277
gi∣291036895∣gb∣GU570163.1∣	*Thanatephorus cucumeris* strain AXN3	99.02	1275
gi∣219972597∣gb∣FJ515881.1∣	*Thanatephorus cucumeris* isolate CHR08-01	100	1275
gi∣326633398∣gb∣JF701746.1∣	*Rhizoctonia solani* isolate RMPM9	100	1267
gi∣291036907∣gb∣GU570171.1∣	*Thanatephorus cucumeris* strain HDY2	98.88	1267
gi∣216963639∣gb∣FJ440211.1∣	*Thanatephorus cucumeris* isolate YWK-208	99.15	1262
gi∣326633385∣gb∣JF701733.1∣	*Rhizoctonia solani *isolate RHRW16	99.71	1260
gi∣326633427∣gb∣JF701775.1∣	*Rhizoctonia solani *isolate RUPU82	98.73	1258
gi∣326633425∣gb∣JF701773.1∣	*Rhizoctonia solani* isolate RUPM83	98.19	1251
gi∣291036890∣gb∣GU570159.1∣	*Thanatephorus cucumeris* strain ACBT13	98.2	1249
gi∣219972641∣gb∣FJ515885.1∣	*Thanatephorus cucumeris* isolate CHR08-14	99.28	1247
gi∣333034239∣gb∣JF429710.1∣	*Thanatephorus cucumeris* strain JS-1	100	1245
gi∣385215144∣gb∣JQ410052.1∣	*Rhizoctonia solani *isolate 0465	100	1243
gi∣534289541∣gb∣KF053536.1∣	*Rhizoctonia solani* isolate GDHY38	99.85	1240
gi∣534289541∣gb∣KF053536.1∣	*Rhizoctonia solani* isolate GDHZ12	100	93.5
gi∣534289540∣gb∣KF053535.1∣	*Thanatephorus cucumeris* genes	99.85	1240
gi∣534289540∣gb∣KF053535.1∣	*Thanatephorus cucumeris* genes	100	93.5
gi∣42475513∣dbj∣AB122135.1∣	*Rhizoctonia solani *	99.85	1236
gi∣326633441∣gb∣JF701789.1∣	*Rhizoctonia solani* isolate Rs9	98.98	1225
gi∣158347474∣gb∣EU152868.1∣	*Rhizoctonia solani* isolate Ms-2	99.41	1223

**Table 4:** The correlation between the average number of lesions and severity index in strain 1801.

		Average total lesion	Severity index
Average total lesions	Pearson correlation	1	0.998∗∗
	Sig. (2-tailed)		0.000
	Number	20	20

Severity index by 1801	Pearson correlation	0.998∗∗	1
	Sig. (2-tailed)	0.000	
	Number	20	20

**Correlation is significant at 0.01 (2-tailed).

**Table 5:** The correlation between the average number of lesions and severity index in strain 1802.

		Average total lesion	Severity index
Average total lesions	Pearson correlation	1	0.996∗∗
	Sig. (2-tailed)		0.000
	Number	20	20

Severity index by 1802	Pearson Correlation	0.996∗∗	1
	Sig. (2-tailed)	0.000	
	Number	20	20

**Correlation is significant at 0.01 (2-tailed).

**Table 6:** Screening of rice varieties with *R. solani* 1801/UPM.

Paddy variety	Country of origin	Resistant∗∗∗∗	Disease extent∗	Scoring scale∗∗	Type∗∗∗
MR219	Malaysia	RB	4	5	MS
IR8	Philippines	BB	3	4	MR
UKMRC2	Malaysia		3	5	MS
IR20	Philippines	BB/RT/RB	5	9	HS
UKMRC9	Malaysia	RB	2	4	MR
TETEP	Vietnam	ShB/RB	2	3	R
Teqing	China	ShB/RB	1	2	R
IR24	Philippines	RS/RB	4	7	S
IR36	Philippines	RT/BLB/RB	4	7	S
IR64	Philippines	RB	3	6	MS
Jasmine85	Philippines	ShB	2	3	R
C4-113	Philippines		5	6	S
ADT 39	India	RB/BLB	3	4	MR
IR50	Philippines	RS	4	5	MS
ADT 38	India	RB/RT	4	7	S
IR39-14	Philippines		3	5	MS
ADT 36	India	RB	3	5	MS
MAHSURI	Malaysia		2	4	MR
TOX 2104-2-1	Nigeria		2	4	MR

*Scored on a scale of 1–5.

∗∗Scored on scale of 1–9 under the standard system evaluation for rice.

∗∗∗HS: highly susceptible; S: susceptible; MS: moderately susceptible; MR: moderately resistant; R: resistant; HR: highly resistant.

∗∗∗∗RB: rice blast; BB: bacterial blight; RT: rice tungro; ShB: sheath blight; BLB: bacterial leaf blight and RS: rice stripe.

**Table 7:** Screening of rice varieties with *R. solani* 1802/KB.

Paddy variety	Country of origin	Resistance∗∗∗∗	Disease extent∗	Scale scoring∗∗	Type∗∗∗
MR219	Malaysia	RB	4	6	S
IR8	Philippines	BB	4	5	MS
UKMRC2	Malaysia		4	6	S
IR20	Philippines	BB/RT/RB	5	9	HS
UKMRC9	Malaysia	RB	3	5	MS
TETEP	Vietnamese	ShB/RB	3	4	MR
Teqing	China	ShB/RB	2	4	MR
IR24	Philippines	RS/RB	4	7	S
IR36	Philippines	RT/BLB/RB	4	7	S
IR64	Philippines	RB	3	6	S
Jasmine85	Philippines	ShB	3	4	MR
C4-113	Philippines		5	6	S
ADT 39	India	RB/BLB	3	5	MS
IR50	Philippines	RS	4	7	S
ADT 38	India	RB/RT	4	7	S
IR39-14	Philippines		3	6	S
ADT 36	India	RB	3	5	MS
MAHSURI	Malaysia		3	5	MS
TOX 2104-2-1	Nigeria		3	5	MS

*Scored on a scale of 1–5.

∗∗Scored on scale of 1–9 under the Standard System Evaluation for Rice.

∗∗∗HS: highly susceptible; S: susceptible; MS: moderately susceptible; MR: moderately resistant; R: resistant; HR: highly resistant.

∗∗∗∗RB: rice blast; BB: bacterial blight; RT: rice tungro; ShB: sheath blight; BLB: bacterial leaf blight and RS: rice stripe.

**Table 8:** Susceptibility index and total lesion length for the top eight cultivars when inoculated with *R. solani* strain 1802.

Cultivar	Susceptibility index		Total lesion length (cm)	
	Percent	Rank	Length	Rank
TOX 2104-2-1	69.5^a^	1	57.40	2
IR8	68.9^a^	2	59.30	1
MAHSURI	56.7^b^	3	43.87	5
UKMRC9	54.8^b^	4	46.60	4
ADT 39	55.6^bc^	5	51.46	3
TETEP	48.8^cd^	6	42.91	6
Jasmine85	43.6^d^	7	29.35	7
Teqing	21.3^e^	8	18.95	8

Mean with different letters displayed in the same column are significant different in the probability of 95 % (*P* < 0.05).

## References

[1] Sneh B, Burpee L, Ogoshi A (1991). *Identification of Rhizoctonia Species*.

[2] Sharon M, Kuninaga S, Hyakumachi M, Naito S, Sneh B (2008). Classification of *Rhizoctonia* spp. using rDNA-ITS sequence analysis supports the genetic basis of the classical anastomosis grouping. *Mycoscience*.

[3] Carling DE, Sneh B, Jabaji-Hare S, Neate S, Dijst G (1996). Grouping in *Rhizoctonia solani* by the anastomosis reaction. *Rhizoctonia Species: Taxonomy, Molecular Biology, Ecology, Pathology, and Disease Control*.

[4] Zheng A, Lin R, Zhang D (2013). The evolution and pathogenic mechanisms of the rice sheath blight pathogen. *Nature Communications*.

[5] Rinehart TA, Copes WE, Toda T, Cubeta MA (2007). Genetic characterization of binucleate *Rhizoctonia* species causing web blight on Azalea in Mississippi and Alabama. *Plant Disease*.

[6] Saitou N, Nei M (1987). The neighbor-joining method: a new method for reconstructing phylogenetic trees. *Molecular Biology and Evolution*.

[7] Rush MC, Lee FN, Webster RK, Gunnell PS (1992). Sheath blight. *Compendium of Rice Diseases*.

[8] Rani DV, Reddy NP, Devi UG (2013). Management of maize banded leaf and sheath blight with fungicides and biocontrol agents. *Annals of Biological Research*.

[9] Parmeter JR, Sherwood RT, Platt WD (1969). Anastomosis grouping among isolates of *Thanatephorus cucumeris*. *Phytopathology*.

[10] Ceresini PC, Shew HD, James TY, Vilgalys RJ, Cubeta MA (2007). Phylogeography of the Solanaceae-infecting Basidiomycota fungus Rhizoctonia solani AG-3 based on sequence analysis of two nuclear DNA loci. *BMC Evolutionary Biology*.

[11] Harikrishnan R, Yang XB (2004). Recovery of anastomosis groups of *Rhizoctonia solani* from different latitudinal positions and influence of temperatures on their growth and survival. *Plant Disease*.

[12] Ciampi MB, Meyer MC, Costa MJN, Zala M, McDonald BA, Ceresini PC (2008). Genetic structure of populations of Rhizoctonia solani anastomosis group-1 IA from soybean in Brazil. *Phytopathology*.

[13] González García V, Portal Onco MA, Rubio Susan V (2006). Review. Biology and systematics of the form genus *Rhizoctonia*. *Spanish Journal of Agricultural Research*.

[14] Grosch R, Schneider JHM, Peth A (2007). Development of a specific PCR assay for the detection of Rhizoctonia solani AG 1-IB using SCAR primers. *Journal of Applied Microbiology*.

[15] Guillemaut C, Edel-Hermann V, Camporota P, Alabouvette C, Richard-Molard M, Steinberg C (2003). Typing of anastomosis groups of *Rhizoctonia solani* by restriction analysis of ribosomal DNA. *Canadian Journal of Microbiology*.

[16] Tamura K, Nei M, Kumar S (2004). Prospects for inferring very large phylogenies by using the neighbor-joining method. *Proceedings of the National Academy of Sciences of the United States of America*.

[17] Woodhall JW, Lees AK, Edwards SG, Jenkinson P (2007). Characterization of *Rhizoctonia solani* from potato in Great Britain. *Plant Pathology*.

[18] Lehtonen MJ, Ahvenniemi P, Wilson PS, German-Kinnari M, Valkonen JPT (2008). Biological diversity of *Rhizoctonia solani* (AG-3) in a northern potato-cultivation environment in Finland. *Plant Pathology*.

[19] Liò P, Goldman N (1998). Review: models of molecular evolution and phylogeny. *Genome Research*.

[20] Kanematsu S, Naito S (1995). Genetic identification of *Rhizoctonia solani* AG 2-3 by analyzing restriction fragment length polymorphisms of nuclear ribosomal DNA internal transcribed spacers. *Annals of the Phytopathological Society of Japan*.

[21] Matsumoto M, Furuya N, Takanami Y, Matsuyama N (1996). A study of fatty acid analysis as a new taxonomic tool for differentiating *Rhizoctonia* spp.. *Journal of the Faculty of Agriculture, Kyushu University*.

[22] Bernardes-de-Assis J, Storari M, Zala M (2009). Genetic structure of populations of the rice-infecting pathogen *Rhizoctonia solani* AG-1 IA from China. *Phytopathology*.

[23] Groth DE (2008). Effects of cultivar resistance and single fungicide application on rice sheath blight, yield, and quality. *Crop Protection*.

[24] Hashiba T (1984). Estimating method of severity and yield loss by rice sheath blight disease. *Bulletin of the Hokuriku National Agricultural Experiment Station*.

[25] Lee FN, Rush MC (1983). Rice sheath blight: a major rice disease. *Plant Disease*.

[26] Park D, Sayler RJ, Hong Y, Nam M, Yang Y (2008). A method for inoculation and evaluation of rice sheath blight disease. *Plant Disease*.

[27] Eizenga GC, Prasad B, Jackson AK, Jia MH (2013). Identification of rice sheath blight and blast quantitative trait loci in two different O. sativa/O. nivara advanced backcross populations. *Molecular Breeding*.

[28] Lakshmanan P (1991). Resistance to sheath blight (ShB) and brown spot (BS) in lines derived from *Oryza officinalis*. *International Rice Research Newsletter*.

[29] Prasad B, Eizenga GC (2008). Rice sheath blight disease resistance identified in *Oryza* spp. accessions. *Plant Disease*.

[30] Pascual CB, Toda T, Raymondo AD, Hyakumachi M (2000). Characterization by conventional techniques and PCR of *Rhizoctonia solani* isolates causing banded leaf sheath blight in maize. *Plant Pathology*.

[31] Pannecoucque J, Van Beneden S, Höfte M (2008). Characterization and pathogenicity of *Rhizoctonia* isolates associated with cauliflower in Belgium. *Plant Pathology*.

[32] Johansson KE, Heldtander MU, Pettersson B (1998). Characterization of mycoplasmas by PCR and sequence analysis with universal 16S rDNA primers. *Methods in Molecular Biology*.

[33] Al-Samarrai TH, Schmid J (2000). A simple method for extraction of fungal genomic DNA. *Letters in Applied Microbiology*.

[34] Bruns TD, White TJ, Taylor JW (1991). Fungal molecular systematics. *Annual Review of Ecology and Systematics*.

[35] Jones DT, Taylor WR, Thornton JM (1992). The rapid generation of mutation data matrices from protein sequences. *Computer Applications in the Biosciences*.

[36] Tamura K, Peterson D, Peterson N, Stecher G, Nei M, Kumar S (2011). MEGA5: molecular evolutionary genetics analysis using maximum likelihood, evolutionary distance, and maximum parsimony methods. *Molecular Biology and Evolution*.

[37] Felsenstein J (1985). Confidence limits on phylogenies: an approach using the bootstrap. *Evolution*.

[38] Tamura K, Nei M (1993). Estimation of the number of nucleotide substitutions in the control region of mitochondrial DNA in humans and chimpanzees. *Molecular Biology and Evolution*.

[39] Willocquet L, Fernandez L, Savary S (2000). Effect of various crop establishment methods practised by Asian farmers on epidemics of rice sheath blight caused by *Rhizoctonia solani*. *Plant Pathology*.

[40] Ogoshi A (1985). Anastomosis and intraspecific groups of *Rhizoctonia solani* and binucleate *Rhizoctonia*. *Fitopatologia Brasileira*.

[41] Ogoshi A (1987). Ecology and pathogenicity of anastomosis and intraspecific groups of *Rhizoctonia solani* Kuhn. *Annual Review of Phytopathology*.

[42] Carling DE, Leiner RH (1986). Isolation and characterization of *Rhizoctonia solani* and binucleate *R. solani*-like fungi from aerial stems and subterranean organs of potato plants. *Phytopathology*.

[43] Pung H, Cross S, Keller KO, McKay A (2007). Investigations on Rhizoctonia solani in cropping soils and vegetable crops. Project HVG05090. *Final Report*.

[44] Kim KH, Rush MC (1986). Inheritance of infection cushion formation by *Rhizoctonia solani* Kühn on rice leaf sheath. *The Korean Journal of Breeding Science*.

[45] Stodart BJ, Harvey PR, Neate SM, Melanson DL, Scott ES (2007). Genetic variation and pathogenicity of anastomosis group 2 isolates of *Rhizoctonia solani* in Australia. *Mycological Research*.

[46] Fang X, Finnegan PM, Barbetti MJ (2013). Wide variation in virulence and genetic diversity of Binucleate *Rhizoctonia* isolates associated with root rot of strawberry in Western Australia. *PLoS ONE*.

[47] Nei M, Kumar S (2000). *Molecular Evolution and Phylogenetics*.

[48] Linde CC, Zala M, Paulraj RSD, McDonald BA, Gnanamanickam SS (2005). Population structure of the rice sheath blight pathogen Rhizoctonia solani AG-1 IA from India. *European Journal of Plant Pathology*.

[49] Butler EE, Lyda SD, Kenerley CM (1993). Rhizoctonia. *Biology of Sclerotial-Forming Fungi*.

[50] Morrison WC (1996). *Louisiana Soybean Handbook*.

[51] Kalpana K, Maruthasalam S, Rajesh T (2006). Engineering sheath blight resistance in elite indica rice cultivars using genes encoding defense proteins. *Plant Science*.

[52] Groth DE (2005). Azoxystrobin rate and timing effects on rice sheath blight incidence and severity and rice grain and milling yields. *Plant Disease*.

[53] Jia Y, Correa-Victoria F, McClung A (2007). Rapid determination of rice cultivar responses to the sheath blight pathogen *Rhizoctonia solani* using a micro-chamber screening method. *Plant Disease*.

