# Supplementary material for: Molecular Characterization and Screening for Sheath Blight Resistance Using Malaysian Isolates of Rhizoctonia solani
Source: Biomed Res Int. 2014 Aug 28;2014:434257. doi: 10.1155/2014/434257 (PMC4166448; doi:10.1155/2014/434257)
Supplement: Supplementary file 1 — The 1801/UPM was amplified using the primer pairs as in Table 1. Primer pairs ITS1/GMRS3 and ITS1/ITS4 successfully amplified the ITS region of this strain. An amplicon of ~550 bp was observed with primers ITS1/GMRS3 (Supplementary File, Figure 1A and 1B) while primers ITS1/ITS4 produced amplicons of ~720 bp (Supplementary File, Figure 2A and 2B). [file 434257.f1.docx]

**Supplementary file**

**Manuscript 434257 
Title "Molecular Characterization and Screening for Sheath Blight Resistance Using Malaysian Isolates of Rhizoctonia solani," by Kalaivani Nadarajah, Nurfarahana Syuhada Omar, Marhamah Md. Rosli and Shin Tze Ong.**

**
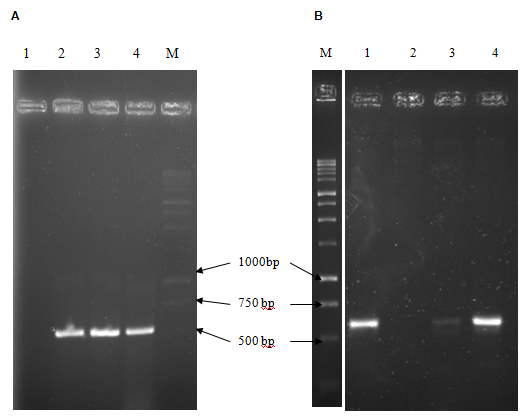
**

**Supplementary File 1A,** PCR products of 1801/UPM strain amplified using ITS1/GMRS3. The single band observed in lane 2-4 is approximately 550 bp. **B,** PCR products of 1802/KB strain amplified using ITS1/GMRS3. The product size is approximately 550 bp.

**
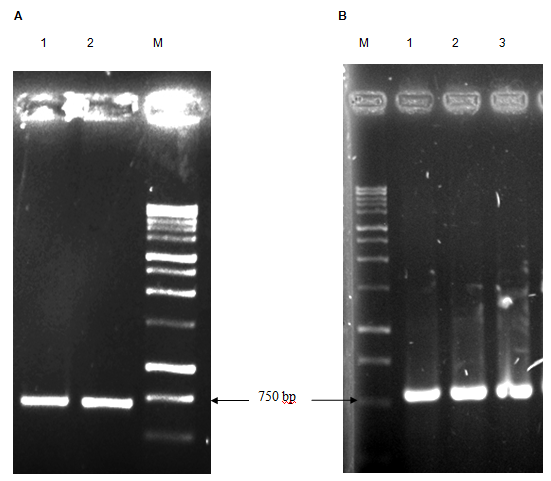
**

**Suplementary File 2A,** Lane M: 1kb DNA ladder (Promega, USA), lane 1-2: PCR products of 1801/UPM strain amplified using ITS1/ITS4. The single band observed in lane 2-3 is approximately 720 bp. **B,** Lane M: 1kb DNA ladder (Promega, USA), lane 1-3: PCR products of 1802/KB strain amplified using ITS1/ITS4. The single band observed in lane 1-3 is approximately 720 bp.
